# The role of ankyrin repeat-containing proteins in epigenetic and transcriptional regulation

**DOI:** 10.1038/s41420-025-02519-4

**Published:** 2025-05-11

**Authors:** Meijuan Wu, Yulu Zhao, Jiahe Yang, Fangyuan Yang, Yeyang Dai, Qian Wang, Cheng Chen, Xiaoyuan Chu

**Affiliations:** 1 Department of Medical Oncology, Nanjing Jinling Hospital, Affiliated Hospital of Medical School, Nanjing University, Nanjing, China; 2 Department of Medical Oncology, Jinling Hospital, Nanjing Medical University, Nanjing, China; 3 Department of Medical Oncology, Jinling Hospital, The First School of Clinical Medicine, Southern Medical University, Nanjing, China

**Keywords:** DNA methylation, Cancer epigenetics

## Abstract

Ankyrin repeat (AR) motif is one of the most abundant repeat motifs found in eukaryotic proteins. It functions in mediating protein–protein interactions and regulating numerous biological functions. Interestingly, some AR-containing proteins are involved in epigenetic and transcriptional events. Our review aims to characterize the structure and post-translational modification of AR, summarize the prominent role of AR-containing proteins in epigenetic and transcriptional events, emphasizing the crucial functions mediated by AR motifs.

## Facts

- AR motif is the most abundant repeat motifs found in eukaryotic proteins.
- AR motif functions in mediating protein-protein interactions and involved in numerous biological functions.
- Epigenetic and transcriptional regulation play crucial roles in regulating gene expression and influencing various biological processes.

## Open questions

- Are AR-containing proteins involved in epigenetic and transcriptional events, such as histone modification, DNA methylation and interacting with transcription factors?
- Does AR motif play crucial roles in the AR-containing protein’s regulation of epigenetic and transcriptional events, and how does AR motif affect these biological processes?

## Introduction

Ankyrin repeat (AR) motif is considered one of the most abundant protein sequence motifs found in eukaryotic proteins [1]. More than 367,000 predicted AR motifs have been found within 68,471 nonredundant proteins as annotated in SMART database. It was first recognized as a repeating sequence in the yeast cell-cycle regulators Swi6p and Cdc10p, as well as in the developmental regulators Notch and LIN-12 of *Drosophila melanogaster* and *Caenorhabditis elegans* [2]. This ~33-amino acid long repeat was eventually named after the cytoskeletal protein Ankyrin that contains 24 tandem ARs [3]. AR motifs are usually implicated in specific protein-protein interactions and are involved in myriad cellular functions [4, 5].

Epigenetic regulations, involving DNA methylation, histone modification, chromatin remodeling and noncoding RNAs, play crucial roles in regulating gene expression. Notably, some AR-containing proteins (ARP) can participate in epigenetic and transcriptional regulation. For example, euchromatin-associated methyltransferases G9a and G9a-like protein (GLP) are AR-containing proteins which can mediate histone methylation [6]. p28^GANK^, an AR-containing protein, has been reported as an oncoprotein and function in suppressing transcriptional activity of NF-κB [7, 8]. Here we focused on some well-documented AR-containing proteins which regulate epigenetic and transcriptional events, mainly through the AR motifs [9, 10], providing new insight into the functional roles of ARP in epigenetic regulation.

## Characterization of AR

### Structure of AR

**Fig. 1:**
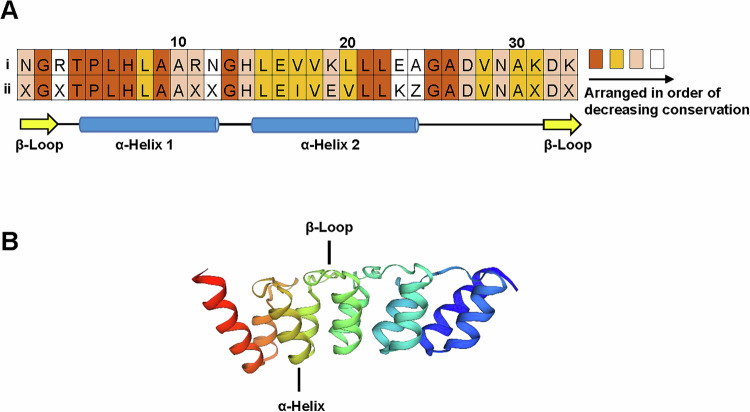
Conserved sequence and structural features of AR motif. **A** Two AR consensus sequences, presented by Mosavi et al. [1] and Kohl et al. [14]. **B** Structure of GABPβ (PDB Q06547) that is made up of AR motifs only.

Each AR motif contains 30-34 amino acids, in which some residues are well conserved to preserve integrity of the motif topology [11]. The consensus sequences of AR are shown in Fig. 1A. Specifically, the T-P-L-H tetrapeptide motif at positions 4-7 is highly prevalent in AR sequences [4, 12, 13]. It forms a tight turn and initiates the first α-helix of an AR motif. The V/I-V-X-L/V-L-L motif (being X any hydrophilic amino acid) is central to the formation of the second α-helix and contributes to the formation of hydrophobic networks within and between AR motifs to stabilize the protein [14]. In addition, glycine residues are conserved at positions 13 and 25, which terminate the helices and provide the freedom for a loop to link both helices [12]. The number of AR motifs can vary from 1 to 34 repeats per protein, with the majority of proteins containing six or fewer repeats [15].

The binding interface of ARs in most of the protein-protein complexes is composed of concave inner surface made up of β-loop region and inner short helices (Fig. 1B). However, the interaction between proteins also happened through the outer helices of ARs called the convex surface [5, 16]. Moreover, many AR-containing proteins undergo structural transitions upon binding [17–19].

### Post-translational modification of AR

A majority of studies has confirmed the presence of post-translational modifications of ARs and we summarize the relatively well-studied ones in the following sections.

#### Hydroxylation

Factor-inhibiting hypoxia-inducible factor (FIH) is well known for catalyzing the β-hydroxylation of an asparagine (Asn) residue in the C-terminal of hypoxia inducible factor (HIF), which suppressed the transcriptional activity of HIF [20, 21]. Kelly et al. first demonstrated that the Asn-hydroxylation on AR could stabilize the localized regions of AR folds [22]. Hardy et al. later confirmed that a single β-hydroxylation of Asn can stabilize the stereotypical AR domain fold, and a second asparaginyl β-hydroxylation caused further stabilization[23]. Following studies found that FIH catalyzed the highly conserved Asn residues within the ubiquitous AR-containing proteins, such as NFκB/IκBα family proteins, Notch receptors, ASB4 and ASPP2 [24–28]. Besides, Yang et al. revealed that the AR of ankyrin-R was multiply hydroxylated by FIH. Hydroxylation of the D34 region (ankyrin repeats 13–24) of ankyrin-R increased its conformational stability and led to a reduction in its interaction with CDB3, demonstrating the potential for FIH-catalyzed hydroxylation to modulate protein-protein interactions. Intriguingly, they found that aspartate (Asp) residues in ankyrin-R and ankyrin-B were also hydroxylated [29].

#### S-palmitoylation

S-palmitoylation is a reversible form of post-translational modification regulating protein attachment to lipid membranes and is catalyzed by palmitoyl acyl transferases (zDHHC PATs) [30]. It is a critical process to stabilize proteins in dendritic spines and dendrites [31], and to localize proteins to different neuronal subcompartments [32].

Ankyrin-G, a member of the ankyrin family, has been reported to be S-palmitoylated at a single cysteine in its N-terminal AR domain [33]. Ankyrin-G has multiple isoforms and the 190 kDa, 270 kDa, and 480 kDa isoforms are the most prominent isoforms in brain [34]. Palmitoylation was known to play an important role in the function of 270 and 480 kDa isoforms [35, 36]. Recently, Nicolas et al. demonstrated that Cys70 palmitoylation in AR domain of 190 kDa isoform of ankyrin-G stabilized its localization in spine heads and at dendritic plasma membrane nanodomains [37]. Another study by Gupta et al. reported that five cysteines in the N-terminal AR domain of ankyrin-B was lipid-modified by S-palmitoylation to promote dendritic localization of the voltage-gated sodium channel Na_v_ 1.2 [38].

#### Phosphorylation

Phosphorylation of ARs has also been reported. Ranganathan et al. demonstrated that casein kinase 2 (CK2) could phosphorylate the serine 1901 (Ser-1901) located in the AR of Notch, which generated a second phosphorylation site at threonine 1898 (Thr-1898). Further studies revealed that Thr-1898 phosphorylation only occurred when Notch formed a complex with Mastermind and CSL. Phosphorylation of both Thr-1898 and Ser-1901 resulted in reduced binding of the Notch-Mastermind-CSL ternary complex to DNA and consequently lowered transcriptional activity of Notch [39]. Besides, cyclin-dependent kinase 5 (Cdk5) has been reported to phosphorylate the ARs of pain-transducing ion channel TRPA1 and modulate its activity. This phosphorylation would occur at characteristic pockets within the (T/S)-P-L-H motifs of AR [40]. Similarly, the ARs of tumor suppressor BARD1 can be phosphorylated by Cdk5, and the T/S-X-X-H motifs of AR were potential substrates for Cdk5 [41].

## AR-containing proteins and epigenetic modifications

### AR-containing histone methyltransferases: G9a and GLP

**Fig. 2:**
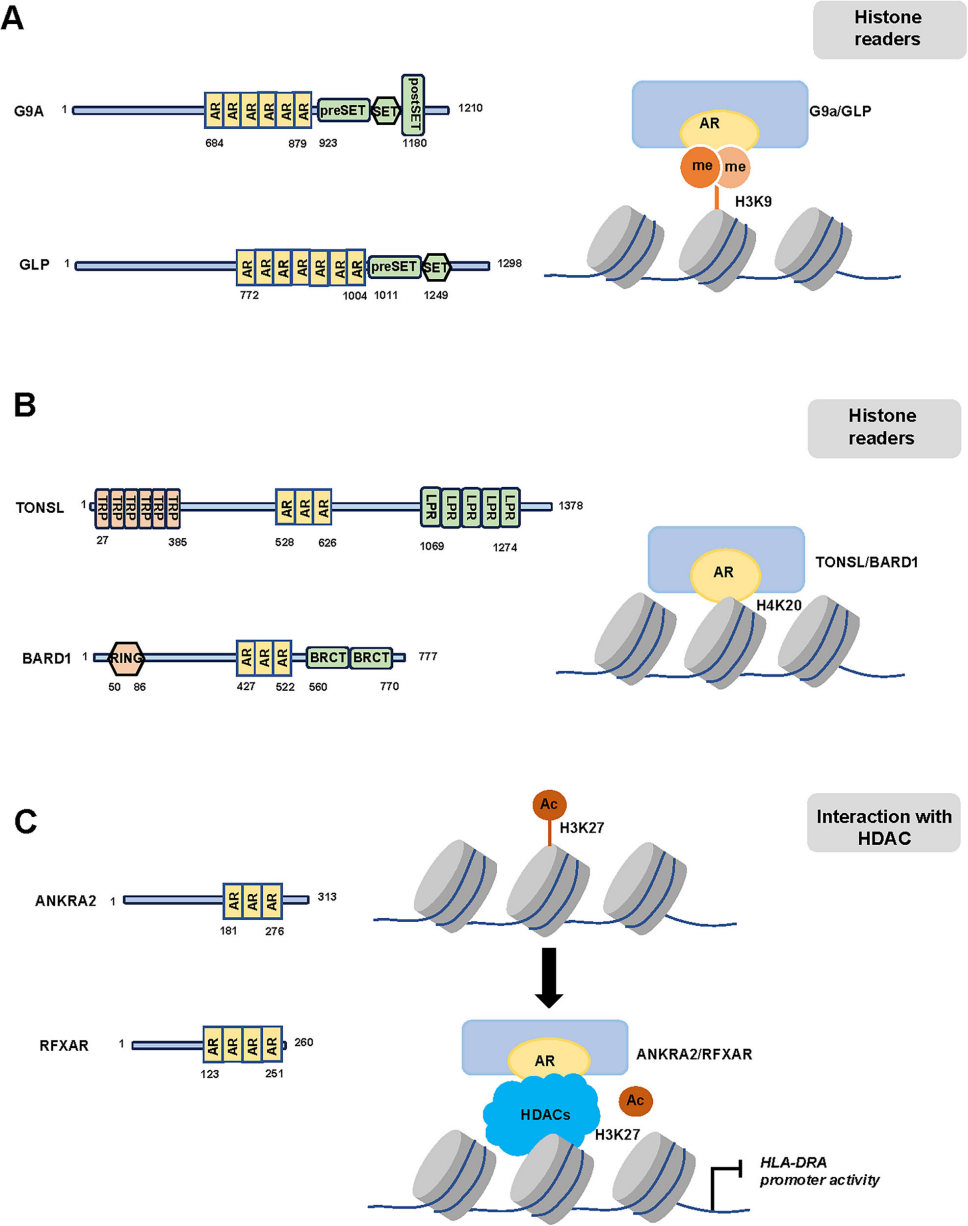
Putative domain architecture of AR-containing proteins involved in epigenetic events. **A** The AR domains of G9a and GLP bound to the H3K9me1/2. **B** The AR domains of TONSL and BARD1 bound to the H4K20me0. **C** The AR domains of ANKRA2 and RFXAR interacted with HDACs to cause histone deacetylation and transcriptional repression.

Histone methylation consists of three important components: histone methyltransferases (writers), histone methylation recognizing proteins (readers), and histone demethylases (erasers). Histone methylation occurs at its arginine or lysine residues, and can either promote or inhibit gene expression, which depends on the specific situation [42]. G9a and G9a-like protein (GLP) are famous euchromatin-associated methyltransferases with AR domain in the N-terminal regions and SET domain in the C-terminal regions (Fig. 2A). G9a and GLP function in transcription repression by mono- and dimethylating histone H3K9 (H3K9me1 and H3K9me2) through the methyltransferase activity of their SET domain [43–45].

Collins et al. demonstrated that the AR domains of G9a and GLP bound with strong preference to H3K9me1 and H3K9me2, which involved recognition of two different features of the methylated histone by distinct regions of AR domains. One is the partial hydrophobic cage of AR recognizing the dimethylamino moiety of H3K9me2, and another is the nearby non-cage functional surface of AR to interact with residues 10-13 of histone H3. G9a/GLP mutants failed to bind methylated H3K9 due to the disruption of their aromatic cages and hydrogen bonds within the AR domain [6]. Bittencourt et al. later established clonal embryonic stem cell (ESC) lines expressing G9a with point mutations in the AR domain that prevent K9-dimethylated histone H3 binding [46]. They confirmed that the Asp905 residue in the AR domain of G9a was critical for maintaining cellular H3K9me2 levels in undifferentiated ESCs.

Apart from functioned as euchromatic histone methyltransferases to catalyze H3K9me1/2, the G9a/GLP complex could also promote gene silencing by inducing DNA hypermethylation in catalytic activity-dependent and -independent manners [47–49]. G9a/GLP functioned as a scaffold to recruit DNA methyltransferase proteins, such as DNMT1, and caused DNA methylation [50]. Bittencourt et al. revealed that the Asp905 residue in G9a’s AR domain was critical for de novo DNA methylation during ESCs differentiation [46].

In addition, AR-containing proteins TONSL [51] and BARD1 [52] have been reported to be readers of H4K20me0 via their AR domains (Fig. 2B). TONSL used its elongated concave surface in AR domain to form extensive contacts with the H4 tail in an extended β-strand-like conformation [51]. Notably, the structure of TONSL AR domain was highly similar to the AR domain of BARD1. BARD1 AR domain mutations disabled H4K20me0 recognition and abrogated accumulation of BRCA1 at genotoxic DNA double-strand breaks. Multiple mutations in the TONSL AR domain have been reported in cancer, including the key histone H4 binding residue, the N571 residue, which corresponded to the BARD1 N470S mutation [53–55]. Altogether, these studies suggested a role of certain AR domains as histone readers.

### AR-containing proteins interacting with HDACs

In histone acetylation, certain lysine residue(s) in the N-terminal tail of histone proteins are acetylated. These reactions are typically catalyzed by histone acetyltransferases and can be antagonized by histone deacetylases (HDACs) [56]. Acetylation of core histones (mainly H3K27ac) leads to chromatin relaxation and transcriptional activation, whereas deacetylation of histones by HDACs is associated with transcriptional repression [57]. In the following section, we summarize some AR-containing proteins that interact with HDACs.

#### ANCO1 (ANKRD11)

Ankyrin repeats-containing cofactor 1(ANCO1, also known as ankyrin repeat domain 11 ANKRD11) is a large ~300 kDa protein that binds and regulates the activity of a number of transcriptional regulators [58]. Zhang et al. demonstrated that ANCOs could bind to the conserved Per-Arnt-Sim region of the p160 coactivators and interact with HDACs, leading to reduced transcriptional activation [59].

Several reports have demonstrated the importance of ANCO-HDAC interaction in breast cancer. Jason et al. showed that the nuclear receptor coactivator AIB1 could recruit ANCO1. The AIB1-ANCO1 complex subsequently recruited HDAC3 and HDAC4 to the intronic estrogen response element, thereby the proximal promoter acquired repressive chromatin mark [60]. Another report demonstrated that ANCO1 worked as a downstream effector of SERPINA3 in mediating endocrine-resistance by interacting with HDAC3 to upregulate its histone deacetylase activity. Thus, decreased SERPINA3 as well as increased ANCO1 expression may predict the efficiency of HDAC3 inhibitor in treating aromatase inhibitor (AI) -resistant breast cancer [61]. Yuan et al. showed that high levels of nuclear ANCO1 predicted more favorable outcomes, especially in the triple-negative breast cancer (TNBC) subtype, and that loss of ANCO1 expression drove early-stage TNBC cells to more malignant phenotypes. They observed a global increase in H3K27ac signals on AP-1, TEAD, STAT3, and NFκB promoters in ANCO1-depleted cells, indicating that ANCO1 may act as a chromatin remodeler in early-stage TNBC [62].

#### ANKRA2 and RFXANK

Ankyrin repeat family A protein 2 (ANKRA2) interacts with the plasma membrane receptors megalin and BK_Ca_ potassium channel [63, 64]. Regulatory factor X–associated ankyrin-containing protein (RFXANK) is homologous to ANKRA2 and positively regulates *MHC II* gene expression [65–68]. Both ANKRA2 and RFXANK contain ARs in the C-terminal domains (Fig. 2C).

McKinesy et al. demonstrated that class II HDACs, HDAC4 and HDAC5, could interact with the ARs of ANKRA2 and RFXANK [57]. Xu et al. further confirmed that the ARs of ANKRA2 and RFXANK could recognize a consensus PxLPxl/L motif found in HDAC4, HDAC5, and HDAC9 in a tumbler-lock binding mode [69]. Specifically, the AR domain of ANKRA2 used an extended binding groove to recognize the PxLPxI/L motif, with three hydrophobic pockets formed by the middle three ARs. Moreover, phosphorylation of Ser^350^ within the consensus motif of HDAC4 (PSLPxI) resulted in reduced binding to ANKRA2, but generated a docking site for 14-3-3 proteins which acted as regulators of the transcriptional repression activities of HDACs. The 14-3-3-binding groove is mostly positively charged, whereas the ANKRA2-binding surface is mostly neutral to negatively charged. This distinctive feature may be the structural determinant of this binary phospho-switch [69].

Altogether, these studies suggested that some AR-containing proteins could interact with HDACs (most likely mediated by ARs) to upregulate the histone deacetylase activity of HDACs, and lead to transcriptional repression.

## AR-containing proteins interacting with transcription factors

### NF-κB signaling

NF-κB transcription factors commonly function as homo- or heterodimers that are formed from five subunits: RelA (p65), RelB, c-Rel, NF-κB1 (p50/p105) and NF-κB2 (p52/p100) [70, 71]. NF-κB transcription factors play crucial roles in regulating numerous biological and pathological processes, such as development of the immune system, inflammation, and oncogenic initiation and progression. Here, we summarize some AR-containing proteins which can interact with NF-ĸB family via AR domain to regulate NF-κB signaling.

#### IκB family

IκB family includes precursor IκB (p100/ IκBδ and p105/ IκBγ) [72], classical IκB (IκBα, IκBβ, and IκBε) [73], and nuclear IκB (Bcl-3, IκB_NS_, IκBζ, and IκBη) [73, 74]. The AR domain of IκBs contains six to eight AR motifs, which has been reported to mediate the direct binding to NF-κB dimers [75–77].

**Fig. 3:**
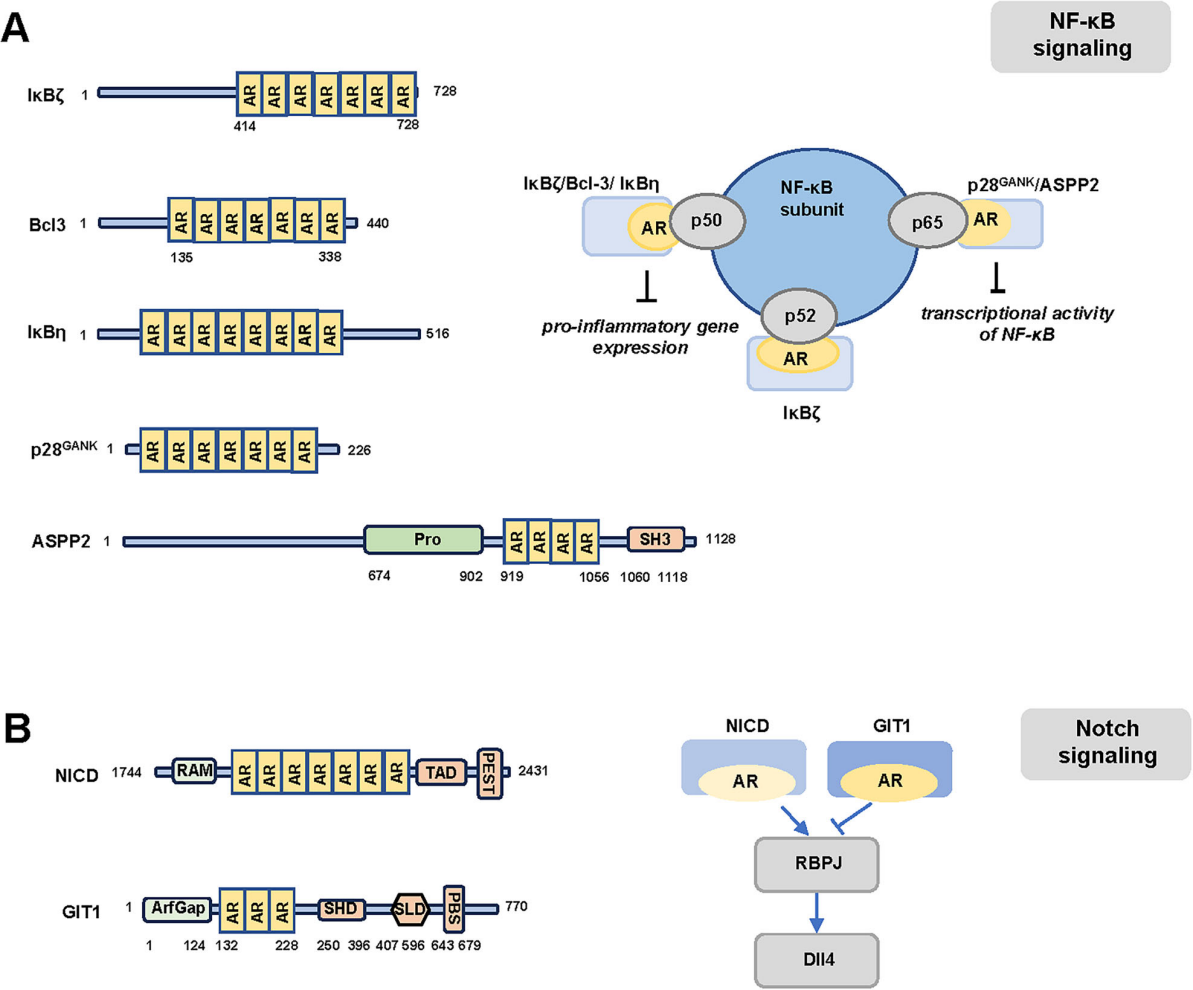
Putative domain architecture of AR-containing proteins involved in transcriptional events. **A** The ARs of IκBζ, Bcl-3, and IκBη bound to NF-κB subunit p50 and inhibited pro-inflammatory gene expression. The ARs of IκBζ could also bind to NF-κB subunit p52. The ARs of p28^GANK^ and ASPP2 bound to NF-κB subunit p65 and inhibited NF-κB transcriptional activity. **B** The ARs of NICD and GIT1 competed for the interaction with RBPJ.

IκBζ, an atypical/nuclear IκB proteins, comprises seven AR motifs in the C-terminal (Fig. 3A). It has been reported to form a transcriptionally active complex with p50/p52 on its target gene *Lcn2* via Asp-451 (located in AR1) [73]. Another nuclear IκB proteins, Bcl-3, has been reported to stabilize a NF-κB p50 homodimer/DNA complex through inhibition of p50 ubiquitination [78]. Further experiments showed that Bcl-3 made extensive contacts with p50 homodimers and in particular with AR 1, 6, and 7, and the N-terminal region of Bcl-3 [79] (Fig. 3A). The direct interaction between p50 and Bcl-3 was required for Bcl-3-mediated inhibition of pro-inflammatory gene expression. Similarly, another nuclear IκB protein, IκBη, has been reported to directly bind to NF-κB. Yamauchi et al demonstrated that IκBη consisted of eight ARs which mediated the interaction with the p50 subunit (Fig. 3A). Besides, the ARs were necessary for the nuclear localization of IκBη [74]. Overall, these studies propose that nuclear IκBs interact with NF-κB subunits via AR motifs.

#### p28^GANK^

p28^GANK^ is an oncoprotein commonly overexpressed in hepatocellular carcinomas [80]. It has been reported to interact with multiple proteins and accelerate degradation of tumor suppressors Rb and p53 [8]. p28^GANK^ consists of seven ARs and is structurally similar to IκBs (Fig. 3A). Higashitsuji et al. demonstrated that p28^GANK^ directly bound to RelA and overexpression of p28^GANK^ suppressed transcriptional activity of NF-κB by modulating acetylation via SIRT1, without affecting the DNA-binding activity or nuclear translocation of RelA [8]. However, another study by Chen et al. found that p28^GANK^ inhibited NF-κB transcriptional and DNA-binding activity. p28^GANK^ directly bound to RelA and exported RelA from nucleus through a chromosomal region maintenance-1 (CRM-1) dependent pathway, resulting in the cytoplasmic retention of RelA. Notably, all the ARs of p28^GANK^ were required for its interaction with RelA and was responsible for suppressing RelA nuclear translocation [7].

#### ASPP proteins

The ASPP proteins are apoptosis regulators: ASPP1 and ASPP2 promote, while iASPP inhibits, p53-dependent apoptosis [81, 82]. All members of the ASPP family share sequence homology in the C-terminal part, which contains the proline rich region followed by four ARs and a SH3 domain [83] (Fig. 3A). The C-terminal ARs and SH3 domain (ANK-SH3) mediate the interactions of the ASPP proteins with major apoptosis regulators. For example, the ANK-SH3 of ASPP2 has been reported to interact with the p65 subunit of NFκB [84] (Fig. 3A). The interaction with p65 was also described for iASPP [85].

#### NF-ĸB family

Notably, the NF-ĸB family itself contains ARs which mediate the interaction with other proteins. It was reported that the ubiquitin ligase KPC1 could bind to the AR domain of NF-ĸB p105, stimulating ubiquitination and limiting proteasomal processing of the p105 precursor to generate p50. Overexpression of KPC1 promoted generation of p50, thereby stimulated the expression of an array of tumor suppressors and the expression of cytokines that attracted the host’s NK cells and macrophages into the tumor, resulting in a strong tumor suppressive effect [86].

### Notch signaling

The Notch protein is a large (300 kDa) single-pass type I transmembrane receptor activated by a regulated intramembrane proteolysis [87]. The intracellular domain of Notch (NICD) is composed of the RAM (RBPJκ-associated molecule) domain, an unstructured linker containing a nuclear localization signal and seven ARs [16] (Fig. 3B). The C-terminus contains PEST motifs, which provide degradation signals [88].

It has been reported that the AR domain of NICD could directly interact with transcription factor RBPJ and activate the transcription of downstream target genes, such as Dll4 [89]. Interestingly, the G-protein-coupled receptor kinase interacting protein-1 (GIT1), which was crucial for sustained VEGF stimulation and modulated endothelial cell migration and proliferation, also contained AR domain and could inhibit the NICD-Dll4 signaling pathway by competing with NICD AR domain for binding to RBPJ in stalk cells [90] (Fig. 3B). Thus, by inhibiting the Dll4-Notch1 positive feedback loop, GIT1 could maintain stalk cell phenotype and vein specification, which was essential for sprouting angiogenesis. The gene encoding Notch-regulated ankyrin repeat protein (NRARP) is one of a small number of core Notch target genes. Jarrett et al. showed that NRARP could act as a negative feedback regulator of Notch responses by directly binding to the Notch intracellular domain1 (NICD1) and forming a complex with RBPJ. The assembly of NRARP-NICD1-RBPJ complexes relied on simultaneous engagement of RBPJ and NICD1, with the three ARs of NRARP extending the Notch1 ankyrin repeat stack [16].

## AR-containing proteins mediating miRNA biogenesis

**Fig. 4:**
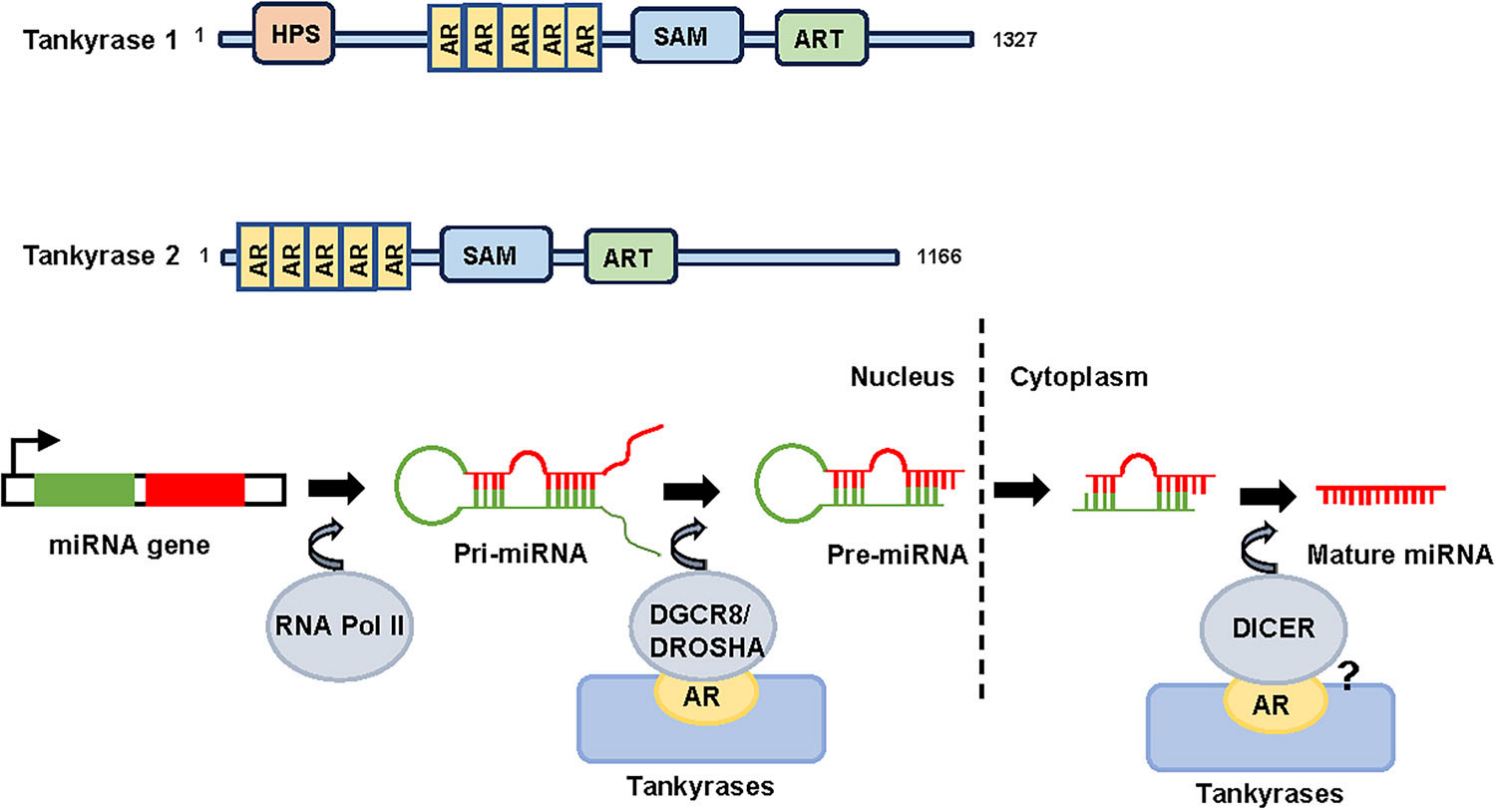
Putative domain architecture of AR-containing proteins involved in miRNA biogenesis. Tankyrase 1/2 bound to DGCR8 and DROSHA via its ARs, promoting pri-miRNA processing to pre-miRNA. Tankyrase 1/2 could also bind to DICER but whether this interaction mediated by ARs remain unknown.

Tankyrases (TNKS1 and TNKS2) are members of poly (ADP-ribose) polymerase (PARP) family proteins [91]. Tankyrases consist of an AR domain, a sterile alpha motif (SAM) and a C-terminal catalytic PARP domain [92] (Fig. 4). The five ARs form conserved ankyrin repeat clusters (ARCs) [93, 94], which recognize the tankyrase-binding motifs in proteins involved in telomere maintenance, Wnt/β-catenin signaling, cancer progression, and DNA damage response [95–100].

MicroRNAs (miRNAs) are critical for post-transcriptional regulation. MicroRNA genes are transcribed as primary miRNAs (pri-miRNAs) by RNA polymerase II (Pol II) in the nucleus [101], and then clopped to precursor miRNAs (pre-miRNAs) by Microprocessor, which comprises DROSHA and DGCR8 [102, 103]. The pre-miRNAs are transferred from nucleus to cytoplasm by exportin 5 (XPO5) and cleaved to mature miRNAs by DICER [104, 105]. Interestingly, many proteins involved in miRNA biogenesis have been reported to have putative tankyrase-binding motifs, such as DROSHA, DGCR8, XPO5, and DICER [106]. Mizutani et al. have recently demonstrated that tankyrase bound to DGCR8 and DROSHA via ARCs, promoting pri-miRNA processing to pre-miRNA [107] (Fig. 4). Similarly, DICER has been reported to bind to tankyrase, but its physiological significance is unknown [108] (Fig. 4). These findings provide new insights into the regulation of miRNA biogenesis mediated by AR-containing proteins. So far, there is no report of AR-containing proteins interaction with lncRNAs or complexes of these molecules, and the possibility of AR-containing proteins in regulating other post transcriptional activities remains to be discovered.

## Conclusions

The biological function of most ARs is to mediate specific protein–protein interactions, and for many of them, the folding of repeats is coupled to the binding of their targets [109]. It is now well documented that folding-upon-binding is crucial for the activity of many AR-containing proteins, including IkB and Notch [19]. The β-hairpins of ARs 5 and 6 in IκBα folded upon binding to NF-κB, but AR 1 remained highly solvent accessible. These regions control degradation of IκBα, enable high-affinity interaction with different NF-κB dimers, and prevent NF-κB from binding to its target DNA. Thus, IκBα conformational flexibility and folding of IκBα upon binding to NF-κB are crucial in regulating NF-κB transcriptional activity [17].

Why do AR motifs in diverse AR-containing proteins exhibit different binding specificities to their partners? We suppose that one reason is that the presence of non-ankyrin domains could affect the orientation of AR domain in the overall 3D structure of the protein [110], which may affect their binding to interactors. Another reason may correlate with the number of ARs. AR-containing proteins with higher number of repeats exhibit a more compact and concave surface of the AR domain [110]. Thus, it is speculated that these proteins exhibit different binding mode from those with lower number of ARs. In addition, the difference in target sequences could also affect binding specificity. For instance, although ANKRA2 and RFXANK are similar in sequence and structure which recognize PxLPxL/I motifs on diverse partners, these two AR-containing proteins exhibit specificity for their partners [69]. Sequences within the PxLPxL/I motifs and the flanking residues determine this specificity.

Overall, our review provides a critical basis for understanding the binding properties of the mentioned AR-containing proteins through AR motifs and their functional relevance to epigenetic and transcriptional events. Expanded biochemical and biophysical examinations are needed to broaden our understanding of how these proteins work and to provide evidence on their potential in clinical applications.

## Data Availability

All data generated or analyzed during this study are included in this published article.
